# Phosphate-Solubilizing Bacteria *Cereibacter sphaeroides* ST16 and ST26 Enhanced Soil Phosphorus Solubility, Rice Growth, and Grain Yield in Acidic-Contaminated Saline Soil

**DOI:** 10.3390/biology14040443

**Published:** 2025-04-19

**Authors:** Le Tien Dat, Le Thi Chinh, Ly Ngoc Thanh Xuan, Le Thanh Quang, Pham Thi Phuong Thao, Do Thi Xuan, Le Thi My Thu, Nguyen Duc Trong, Tran Trong Khoi Nguyen, Nguyen Quoc Khuong

**Affiliations:** 1 Faculty of Crop Science, College of Agriculture, Can Tho University, Can Tho 94000, Vietnam; letiendat84@gmail.com (L.T.D.); lethi.chinh69@gmail.com (L.T.C.); ltquang@ctu.edu.vn (L.T.Q.); thule@ctu.edu.vn (L.T.M.T.); ndtrong@ctu.edu.vn (N.D.T.); ttknguyen@ctu.edu.vn (T.T.K.N.); 2 Branch of Planting & Plant Protection of Agriculture and Rural Development of Vinh Long Province, Vinh Long 85000, Vietnam; 3 Experimental and Practical Area, An Giang University, Vietnam National University Ho Chi Minh City, Long Xuyen 90100, Vietnam; lntxuan@agu.edu.vn; 4 Faculty of Physiology-Biochemistry, College of Agriculture, Can Tho University, Can Tho 94000, Vietnam; ptpthao@ctu.edu.vn; 5 Institute of Food and Biotechnology, Can Tho University, Can Tho 94000, Vietnam; dtxuan@ctu.edu.vn

**Keywords:** phosphate-solubilizing bacteria, purple nonsulfur bacteria, rice plants, saline soil, salinity, salt tolerance

## Abstract

Soil phosphorus is heavily restricted by soil acidification and salinization. There is a need to determine a biological solution for this issue to replace the overuse of chemical phosphorus fertilizer that aggravates adverse conditions, such as salinity, acidity, and metallic toxicity. Therefore, this study aimed at determining the phosphorus dynamics in terms of the soil, growth, and yield of rice under the supplementation of phosphate (P)-solubilizing purple nonsulfur bacteria (PNSB), *Cereibacter sphaeroides* ST16 and ST26, in salinized soil collected from An Bien district, Kien Giang province, Vietnam, under greenhouse conditions. The experiment followed a completely randomized block design with two factors and four replications. In particular, the reduced percentages of P fertilizer (A) were 0%, 25%, 50%, 75%, and 100% P. The supplementations of *C. sphaeroides* strains (B) were the negative control, ST16, ST26, and a mixture of both ST16 and ST26. The results showed that supplying the *C. sphaeroides* ST16 and ST26 reduced the insoluble P content by 10.1–10.6% Fe-P, 10.3–12.2% Ca-P, and 12.7–43.1% Al-P and increased available P by 8.33–27.8%, leading to total P uptake in plants increasing by 29.4–56.1%. The *C. sphaeroides* strains also reduced soil Na^+^. Therefore, supplying the *C. sphaeroides* strains increased the rice growth and yield components of rice, leading to a greater yield of 26.5–51.0%. Supplying each strain of ST16 and ST26 reduced 50–100% P fertilizer as recommended. Ultimately, inoculation of the bacterial mixture allowed a reduction by 100% P fertilizer percentage as recommended but the yield remained the still.

## Simple Summary

Agriculture of the coastal areas in the Vietnamese Mekong Delta is threatened by acidification and salinization. Chemical approaches have been used to improve soil conditions. However, these approaches are unsustainable and not safe for the environment. Thus, biological approaches are preferable. In the current study, a biofertilizer of two strains of *Cereibacter sphaeroides*, ST16 and ST26, was used to improve rice growth under a saline acidic condition in a Vietnamese coastal region in a greenhouse experiment. In particular, the soil health and rice growth were improved under the supplementation of the bacteria. Inoculation of the bacterial mixture allowed a reduction of 50–100% of chemical fertilizer. The current study provided a great candidate to contribute to agricultural environmental remediations and sustainable agriculture. This newly developed biofertilizer should be further tested under field trials.

## 1. Introduction

The Mekong Delta nationally accounts for 50% of rice production and 95% of the exported products in Vietnam [1]. The Mekong Delta has been severely affected by salinization [2,3]. The sea level is predicted to rise by 1 m and cover 45% of the area of the Mekong Delta, in which Kien Giang province will have 76% of its area suffering from salinization, further compounding the problem if there are no adaptation measures [4,5]. To adapt to the salinization in agricultural production, farmers have applied various approaches such as crop diversification, season arrangement to make use of freshwater, genomic diversification to create saline-tolerant crops, organic fertilizer use, and biological use control [5,6,7].

Because rice is greatly vulnerable to high concentrations of Na^+^ and Cl^−^, rice production is low during the dry season [8]. Therefore, rice farming undergoes a rice-shrimp integrating model, especially in coastal regions. This model can bring better profit because shrimps are farmed in the dry season and rice is farmed in the wet season, leading to efficient income throughout the year [7]. However, farming rice in saline soils for a long time can lead to salt accumulation in the soil. Thus, to ease this situation, calcium salt is normally used to reduce salinity [9,10]. Furthermore, approaches using microbial products are considered environmentally friendly [10] because microbial communities’ habitat in the soil plays important roles in nutrient dynamics, organic degradation, crop yield maintenance [11], crop tolerance against adverse conditions, and secondary metabolite production [10].

Therein, purple nonsulfur bacteria (PNSB) are promising because they can live under adverse conditions, such as saline, acidic, and acidic–saline [12,13,14,15,16]. PNSB can improve the fertility of salt-affected soils, with the ability to immobilize Na^+^ by functional groups, such as carboxyl, hydroxyl, and amide in exopolymeric substances produced by PNSB [15]. These bacteria can also solubilize P and produce plant growth-promoting substances (PGPS) such as indole-3-acetic acid (IAA), 5-aminolevulinic acid (ALA), exopolymeric substances (EPS), and siderophores [12]. Moreover, PNSB can live under conditions contaminated with H^+^, Al^3+^, Fe^2+^, and Mn^2+^ [12]. Therefore, combining PGPS production and bioremediation to address causing factors for abiotic stresses is a promising green agricultural approach [10]. Therefore, due to the above characteristics, PNSB has been applied for crops in different types of soil [16] and rice in acidic soil [17]. Therefore, due to the diverse conditions, more and more PNSB candidates have been isolated [18]. Among them, two strains of PNSB, *Cereibacter sphaeroides* ST16 and ST26, have been isolated from paddy fields and showed promising P-solubilizer properties under acidic-salinized conditions [19]. However, these strains of PNSB P-fixers have not been tested to perform on acidic salinized paddy soil. Therefore, the study aimed to investigate the effects of a biofertilizer containing phosphate-solubilizing PNSB strains *Cereibacter sphaeroides* ST16 and ST26 on the soil-available phosphorus nutrients, growth, and yield of rice in acidic-salinized soil under greenhouse conditions.

## 2. Materials and Methods

### 2.1. Experimental Design

The experiment was conducted in the greenhouse of the Agricultural Research and Experiment Camp, College of Agriculture, Can Tho University [10°01′43.2″ N 105°45′58.9″ E]. The conditions of the greenhouse were 37.8 °C in temperature, 60.3% in moisture, and 11/13 h in day/night hours in season 1, while those in season 2 were 34.5 °C, 65.1%, and 11.5/12.5, respectively. The experiment followed a completely randomized block design with four replications. The first factor was the P fertilization at rates of 0% P, 25% P, 50% P, 75% P, and 100% P as compared to the recommendation. The other factor was the *C. sphaeroides* application, including (i) the negative control without bacteria, (ii) *C. sphaeroides* ST16, (iii) *C. sphaeroides* ST26, and (iv) a mixture of both *C. sphaeroides* ST16 and ST26 [19]. Given the combination of 5 levels of fertilizers and 4 levels of bacteria, the experiment included 20 treatments replicated 4 times. Each replication was presented as a block in which 20 treatments were randomly arranged.

The experiment was repeated twice in two continuous seasons to confirm the efficacy of the current biofertilizers. In other words, in the first season, the rice was planted on acidic saline soil, treated according to the experimental design, and harvested. Then, in the second season, new plants were planted in the soil from season 1, treated according to the experimental design, and harvested.

The soil was collected in Hamlet 6, Nam Thai commune, An Bien district, Kien Giang province, Vietnam, at a depth of 0–30 cm (Table 1). The collected soil was prepared by being cleaned from plant residues by a sieve, ground with a mortar and pestle, and dried naturally. In total, 8 kg of the prepared soil and 5 L of tap water were added to a pot, whose size was 23–17–18 cm according to the top–bottom–height, and mixed well.

The two *C. sphaeroides* strains, ST16 and ST26, can solubilize insoluble P compounds (Al-P, Fe-P, and Ca-P) and were isolated from the soil and water in salinized soil in a rice shrimp system [19]. PNSB were propagated according to Khuong et al. [24].

The rice cultivar *Oryza sativa* cv. OM 5451 (Jasmine 85 × OM 2490) was used in the current study. The cultivar has a life span of 88–93 days, 85–95 cm height, 25–26 g per 1000 grains, and a potential yield of 6.00–8.00 t ha^−1^ [25].

The rice grains were sterilized with ethanol 70% (Xilong, Guangzhou, China) and sodium hypochlorite 1% (Xilong, Guangzhou, China) for 10 min, rinsed with distilled water, and incubated for 24 h [12]. Then, the incubated grains were divided into equal portions to be soaked into each of the following solutions: (i) the negative control with only distilled water, (ii) the suspension of *C. sphaeroides* ST16, (iii) the suspension of *C. sphaeroides* ST26, and (iv) the mixture of *C. sphaeroides* ST16 and ST26 suspensions. The bacterial density of each suspension was 1 × 10^8^ CFU mL^−1^. The grain-solution mixture was shaken at 60 rpm for 1 h and let dry under a laminar airflow for 1 h. The grains were subsequently sown into the soil. During the rice growth, 4 mL pot^−1^ of PNSB suspension was supplied on days 7, 14, 21, 28, 35, and 42 after sowing, equivalent to 4 × 10^4^ CFU g^−1^ dry soil.

The NPK fertilizer for rice was 90N–60P_2_O_5_–30K_2_O, which was equivalent to the number of fertilizers of N fertilizer (urea, Phu My company, Phu My, Binh Phuoc province, Vietnam), P fertilizer (superphosphate, Long Thanh company, Long Thanh, Dong Nai province, Vietnam), and K fertilizer (potassium, Phu My company, Phu My, Binh Phuoc province, Vietnam) (195.7, 375, and 50 kg, respectively) for 2,000,000 kg soil per ha [24]. Theoretically, for 8 kg of soil, the required amounts of urea, superphosphate, and potassium fertilizers were 0.78 g, 1.0 g, and 0.2 g, respectively. Overall, 100% of P fertilizer was fertilized before sowing, and N fertilizer was used at the rates of 30, 30, and 40% on days 10, 20, and 45 after sowing. K fertilizer was used at the rates of 50% on days l0 and 45 after sowing. In total, 10 mL of NaCl 4 ‰ (Xilong, Guangzhou, China) was supplied to each rice pot on days 20, 40, and 75 after sowing [19]. The water level in each pot was maintained weekly at 3 cm from the soil by tap water. However, from day 0 to 10 after sowing and day 10 to harvest, the soil was kept at a moderate humidity.

### 2.2. Parameters of Evaluation

#### 2.2.1. Soil Analysis

Methods of Sparks et al. [26] were followed. pH_H2O_ and EC were extracted by distilled water, and pH_KCl_ was extracted by KCl 1.0 M (1 soil: 5 solvent). The extracts of pH_H2O_ and pH_KCl_ were measured by a pH meter (F-73, Horiba, Kyoto, Japan), and EC was measured by an EC meter (D-72A-S, Horiba, Kyoto, Japan). The N_total_ in the soil was measured by the Kjeldahl method in which the soil was digested by the mixture of sulfuric acid-salicylic catalyzed by CuSO_4_: Na_2_SO_4_: Se. The NH_4_^+^ concentration was measured by spectrophotometry (UV/Vis UV1800, Shimadzu, Kyoto, Japan) at a 650 nm wavelength from soil extracted by KCl 2.0 M. The P_total_ was measured from soil digested by saturated H_2_SO_4_–HClO_4_. P_soluble_ in the soil was extracted according to the Bray II method. The insoluble P compounds were measured from soil extracted by NH_4_F 0.5 M (pH = 8.2) for Al-P, NaOH 0.1 M for Fe-P, and H_2_SO_4_ 2.5 M for Ca-P. The three types of insoluble P compounds were rinsed with saturated NaCl twice. All P fractions were measured by spectrophotometry at 880 nm wavelength. The CEC was measured from soil extracted by MgSO_4_ 0.02 M and titrated by EDTA 0.01 M. Cations (K^+^, Na^+^, Ca^2+^, and Mg^2+^) were measured by atomic absorption spectrophotometry at 766, 589, 422.7, and 285.5 nm wavelengths, respectively, from soil extracted by BaCl_2_ 0.1 M.

#### 2.2.2. Plant Analysis

The chlorophyll content in leaves was measured by Chlorophyll Meter SPAD (SPAD-502 Plus, Konica Minolta, Tokyo, Japan) on days 21, 28, 35, and 42 after sowing. The chlorophyll a and b in leaves were measured by spectrophotometry at 664 and 647 nm wavelengths, respectively, on day 45 after sowing [27].

The proline content in the stem–leaf on day 45 after sowing was measured according to Bates et al. [28]. In brief, fresh stems and leaves were milled. In total, 0.5 g of the milled stem–leaf was left to react with 10 mL of sulfosalicylic acid 3% and shaken for 30 min before centrifugation. Then, 2.0 mL of the sample was left to react with 2.0 mL of ninhydrin and 2.0 mL of glacial acetic acid and heated for 1 h. The mixture was then cooled at room temperature, left to react with 4.0 mL of toluene, shaken, and measured by spectrophotometry at 520 nm wavelength.

The agronomic traits were measured according to the description by IRRI [29] for plant height, pencil length, number of panicles pot^−1^, number of grains per panicle, filled grain rate per panicle, yield, and 1000-grain weight. After harvesting, the yield was converted to the yield at a humidity of 14%.

The biomass of stem–leaf and grain after harvesting was dried for 72 h at 70 °C. The dry stem–leaf and grain were milled by a 0.5 mm sieve to analyze P and Na according to Houba et al. [30]. The P content was measured by the ascorbic acid method and spectrophotometry at 880 nm wavelength. The Na content was measured by atomic absorption spectrophotometry at 589 nm wavelength.

### 2.3. Statistical Analysis

SPSS 16.0 was used to compare means between treatments according to the Duncan test at 5% significance.

## 3. Results

### 3.1. Features of the Salinized Soil in the Rice-Shrimp Model in An Bien, An Giang

The soil in the current study was classified as saline acidic soil (Table 1). In particular, pH_H2O_, pH_KCl_, and EC were 3.82, 3.69, and 6.59 mS cm^−1^, respectively. The total N, NH_4_^+^, total P, and PO_4_^3−^ were 0.196%, 49.8 mg kg^−1^, 0.021%, and 85.0 mg kg^−1^, respectively. The Al-P, Fe-P, and Ca-P corresponded to 14.5 mg kg^−1^, 105.3 mg kg^−1^, and 85.0 mg kg^−1^. The CEC was 9.78 meq 100 g^−1^. The concentrations of cations were 3.02 Na^+^, 0.683 K^+^, 3.07 Ca^2+^, and 2.82 Mg^2+^ meq 100 g^−1^.

### 3.2. Cereibacter sphaeroides Affected the Rice Biochemistry

Table 2 provides the individual effects of each factor on rice biochemistry. In Table 2, among P fertilizer rates 0%, 25%, 50%, 75%, and 100% P, SPAD indices significantly varied (37.0–39.8) in the first season. However, the treatments with P fertilizer rates from 50% P to 100% P resulted in greater SPAD than the negative control, with 35.6–36.3 compared with 34.7, respectively, on day 42 after sowing in the second season. Moreover, only the ST26 strain improved the SPAD index in the first season, while all of the applications of ST16 and ST26 resulted in greater SPAD indices (35.1–36.6) compared with the negative control (34.4) on day 42 after sowing in the second season.

The chlorophyll a content increased while the chlorophyll b concentration decreased with P application when compared with no P fertilizer case in both seasons. Furthermore, applying each strain of *C. sphaeroides* ST16 and ST26 or their mixture followed the same trend as the increase in P fertilizer rates for chlorophyll a and b values. The chlorophyll a+b ranged from 6.46 to 8.15 in the first season and 5.41–5.72 in the second season for both factors (Table 2).

Table 2 shows that fertilizing P or applying PNSB decreased proline content in stem–leaf by 3.09–5.00% and 6.20–11.8%, respectively, in both seasons, except for supplying ST16 in the first season and fertilizing 25, 50, and 75% P in the second season. The interactions between the two factors were significant for SPAD on days 21 and 42 after sowing in the second season and on day 35 after sowing in both seasons, for the chlorophyll b and a + b in both seasons, and proline in the second season.

### 3.3. Cereibacter sphaeroides Affected P Dynamics and Soil Features

Table 3 indicates the individual effects of each factor on soil traits. Table 3 shows that pH_H2O_ and pH_KCl_, when supplied with or without P fertilizer, did not statistically change and ranged from 4.51 to 5.64 and from 3.54 to 4.07 in both seasons. The EC increased as compared with the negative control when supplying P from 25% to 100% and ranged from 4.35 to 5.00 mS cm^−1^ in both seasons. Supplying PNSB resulted in increased pH_H2O_ from 4.23 to 4.50–4.98 in the first season and from 5.27 to 5.45–5.83 in the second season and in decreased EC by 15.3% and 21.7% in both seasons. There were significant interactions between the two factors for EC in the second season.

The total N and P did not change under the P fertilization and PNSB supplementation in both seasons. Furthermore, the concentrations of NH_4_^+^, soluble P, Fe-P, Ca-P, and Al-P corresponded to ranges of 115.3–244.1, 4.64–31.2, 115.9–167.0, 62.0–193.5, and 11.7–158.0 mg kg^−1^, respectively, and increased according to P fertilizer rates 0% < 25% < 50% < 75% < 100% P in both seasons. Supplying each strain of *C. sphaeroides* ST16 and ST26 or their mixture increased the NH_4_^+^ concentration by 4.60% and 11.9% and soluble P by 27.8% and 8.33% in both seasons, respectively. The interactions between the two factors differed at 5% significance in NH_4_^+^, soluble P, and insoluble P in both seasons (Table 3).

CEC was equivalent among treatments and ranged from 16.0 to 19.2 meq 100 g^−1^ in both seasons. Furthermore, PNSB improved the concentrations of K^+^ and Ca^2+^ in the soil in both seasons and Mg^2+^ in the second season, except for Mg^2+^ at 25% P as compared with the negative control. The Na^+^ concentration was reduced by 5.87% and 50.0% in both seasons, respectively. Supplying P changed the cation concentrations in the soil. In particular, P fertilization improved K^+^ concentration in the soil in both seasons, reduced Mg^2+^ concentration in the first season but increased Mg^2+^ concentration in the second season, increased Ca^2+^ concentration in the second season, and reduced Na^+^ concentration in both seasons. The interactions between the two factors were significant at 5% between the two factors in concentrations of Na^+^ and K^+^ in both seasons and Mg^2+^ in the second season (Table 3).

### 3.4. Cereibacter sphaeroides Affected P and Na Uptake in Rice Plants

Table 4 indicates the individual effects of each factor on Na and P uptake in rice plants. Table 4 shows that the treatments with both P fertilizers and PNSB had dry biomasses of stem–leaf and grain ranging from 12.0 to 24.9 and from 11.8 to 24.6 g pot^−1^, respectively, and increased according to 0% < 25% < 50% < 75% < 100% P and *C. sphaeroides* ST16 < *C. sphaeroides* ST26 < *C. sphaeroides* ST16 and ST26 in both seasons. The interactions were significant between the two factors.

In Table 4, P fertilization resulted in variations in P contents in stem–leaf and grain. In particular, the P content in stem–leaf (0.140–0.174%) increased compared with the negative control in the second season but varied extremely in the first season. Supplying PNSB also caused variation in P contents in stem–leaf and grain, ranging from 0.138 to 0.193 and from 0.135 to 0.262%, respectively. Supplying each strain or the mixture of PNSB increased the P content in stem–leaf and grain compared with the negative control, except for supplementations of *C. sphaeroides* ST16 or the *C. sphaeroides* mixture in stem–leaf in the first season. The total P uptake (59.0–79.2 mg P pot^−1^) proportionally increased according to the 0% < 25% < 50% < 75% < 100% P in the second season and 0~25~50% < 75% < 100% P in the first season. The P fertilization and PNSB supplementation significantly interacted with each other in P contents in stem–leaf and grain, P uptake in stem–leaf and grain, and total P uptake.

**Figure 1:**
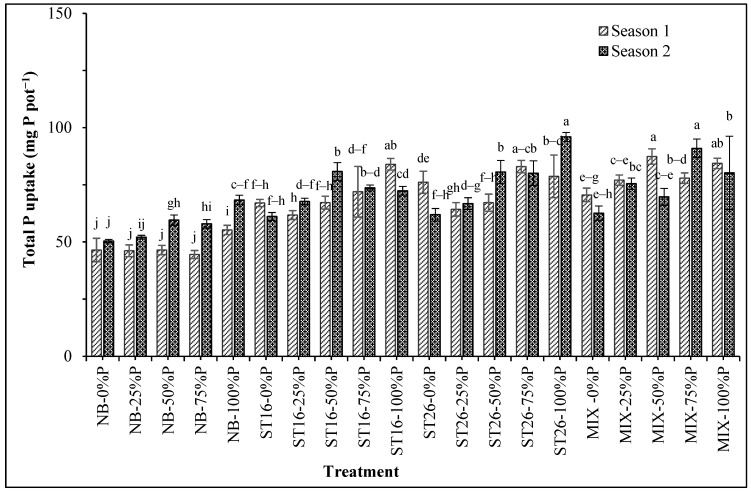
Phosphate fertilization and phosphate-solubilizing *Cereibacter sphaeroides* affected the total P uptake in rice plants in salinized soil in An Bien district, Kien Giang province. Note: 0, 25, 50, 75, 100: P fertilizer percentage; NB: No bacteria; ST16: *Cereibacter sphaeroides* ST16; ST26: *Cereibacter sphaeroides* ST26; MIX: *Cereibacter sphaeroides* ST16 and ST26. Different letters above the bars indicate significant differences among treatments at 5%.

Specifically, the total P uptake increased by 56.1% in the first season and 29.4% in the second season when supplying PNSB. Noticeably, the total P uptake peaked in the treatment with the mixture of the two bacterial strains in both seasons compared to the treatments without PNSB (Table 4). In Figure 1, the combined effects of the two factors on total P uptake are shown. The total P uptake in the treatments with both PNSB and P fertilizer rates from 0% to 100% P in the first season was greater than in the treatment with only 100% P. Supplying the mixture and 50% P in the first season or 75% P in the second season resulted in the greatest total P uptake (Figure 1).

**Figure 2:**
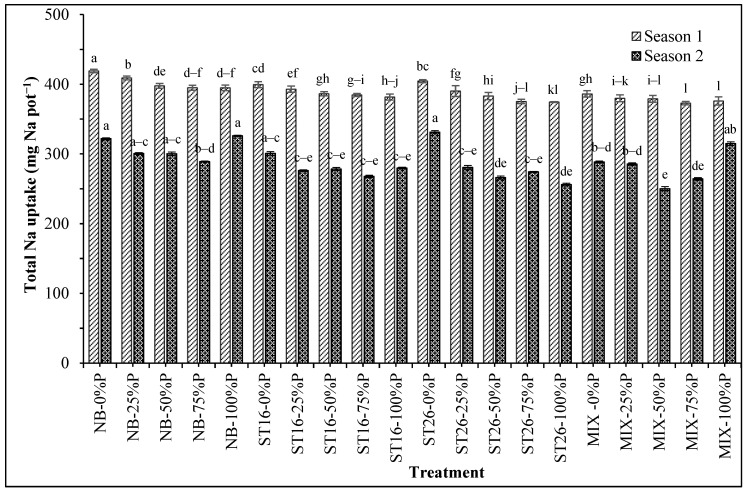
Phosphate fertilization and phosphate-solubilizing *Cereibacter sphaeroides* affected the total Na uptake in rice plants in salinized soil in An Bien district, Kien Giang province. Note: 0, 25, 50, 75, 100: P fertilizer percentage; NB: No bacteria; ST16: *Cereibacter sphaeroides* ST16; ST26: *Cereibacter sphaeroides* ST26; MIX: *Cereibacter sphaeroides* ST16 and ST26. Different letters above the bars indicate significant differences among treatments at 5%.

Table 4 shows that supplying PNSB or P fertilizer decreased the total Na uptake compared with the negative control in both seasons. Figure 2 illustrates the combined effects of the two factors on total Na uptake. The treatment fertilized with P and with or without PNSB resulted in decreased total Na uptake according to the increase in the P fertilizer rate from 0% to 100% P in the first season. Supplying both the mixture of *C. sphaeroides* ST16 and ST26 without P fertilizer resulted in lower total Na uptake than the treatment with only 100% P. In the second season, the total Na uptake varied. The total Na uptake peaked in the treatment with only 100% P. Both supplying the *C. sphaeroides* ST26 strain and P fertilizer from 25% to 100% P resulted in lower total P uptake than the treatment with 100% P (Figure 2).

### 3.5. Cereibacter sphaeroides Affected Rice Agronomic Traits

Table 5 shows the individual effects of each factor on rice performance in salinized soil. Rice plant height was improved when supplied with either P fertilizer or PNSB. In particular, plant height reached 86.8–96.2 cm, which was increased by 4.76% and 2.13% by the P fertilization and by 8.26% and 2.81% by the PNSB in the first and second seasons, respectively. Furthermore, P fertilizer did not affect the panicle length in both seasons. However, the panicle length in the treatment with PNSB was greater than in the negative control in the first season but equivalent to the negative control in the second season. Supplying the *C. sphaeroides* strains resulted in a panicle length of 20.7–20.9 cm in the first season and 18.7–19.2 cm in the second season (Table 5).

Fertilizing P improved the number of panicles pot^−1^ and filled grain percentage, with 20.2–23.0 panicles and 67.2–77.5% compared with 18.3 panicles and 66.2% in the first season; 19.0–21.4 panicles and 90.5–92.5% compared with 18.5 panicles and 89.4% in the second season as compared with the negative control. However, the 1000-grain weight was equivalent among treatments. Supplying PNSB improved the number of panicles pot^−1^, the number grains panicle^−1^, and filled grain percentage, with 20.5–23.6 compared with 16.9 panicles, 75.2–80.0 compared with 60.1 grains, and 70.8–75.2 compared with 66.4% in the first season as well as 19.6–21.5 compared with 16.9 panicles, 65.4–66.3 compared with 63.6 grains, and 90.8–94.1 compared with 87.4% in the second season. However, the 1000-grain weight remained statistically in both seasons (Table 5).

The average rice grain yield increased by 20.7% and 36.5% in the first season and by 15.7% and 15.2% in the second season, according to the P fertilizer rates and PNSB supplementation. The interactions between factors were significant at 5% in plant height, the number of panicles pot^−1^, filled grain percentage, and rice grain yield in both seasons (Table 5).

**Figure 3:**
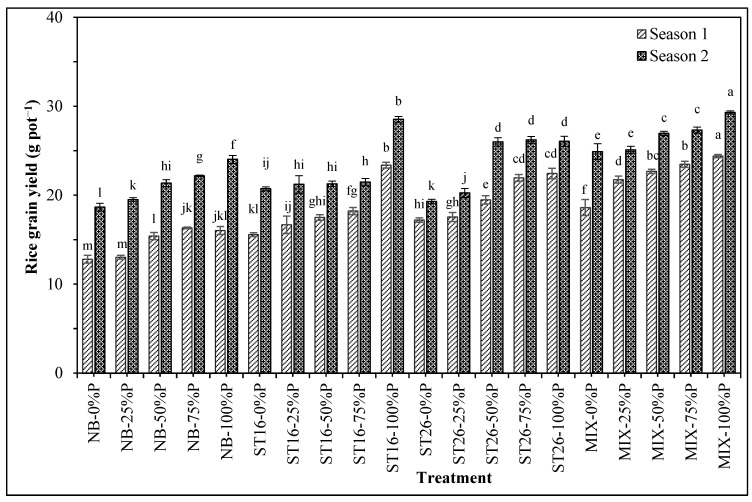
Phosphate fertilization and phosphate-solubilizing *Cereibacter sphaeroides* affected the grain yield of rice in salinized soil in An Bien district, Kien Giang province. Note: NB: No bacteria; ST16: *Cereibacter sphaeroides* ST16; ST26: *Cereibacter sphaeroides* ST26; MIX: *Cereibacter sphaeroides* ST16 and ST26; 0, 25, 50, 75, 100: P fertilizer percentage. Different letters above the bars indicate significant differences among treatments at 5%.

Figure 3 shows the combined effects of the two factors on rice grain yield. In detail, Table 5 and Figure 3 indicate that the grain yield contained interactions between the P fertilizer and PNSB. The yield among P fertilizer percentages and PNSB supplementations increased according to the following order 0% < 25% < 50% < 75% < 100% P (2.87–34.2%) and *C. sphaeroides* ST16 < *C. sphaeroides* ST26 < the mixture of *C. sphaeroides* ST16 and ST26 (7.11–51.0%). Furthermore, supplying both the ST16 and 25% resulted in an equivalent yield to the treatment with only 100% P in the first season. Interestingly, supplying the *C. sphaeroides* ST26 or the PNSB mixture without P fertilization resulted in greater yield than fertilizing with only 100% P in the first season. The yield in the treatments with the PNSB mixture and 0% P was greater than that in the treatment with only 100% P in the second season.

## 4. Discussion

Table 1 reveals that the soil in the current study lacks total P with greater contents of insoluble P. Thus, strains of P-solubilizing bacteria were used to provide available P for rice plants. Some PNSB strains can solubilize P in paddy acidic and saline soils [12,24]. This means indigenous bacteria isolated from rice-shrimp soil should be applied in rice-shrimp soils due to their native adaptability. Hence, supplying *C. sphaeroides* ST16 and ST26 improved soil pH_H2O_. In addition, results of EC, NH_4_^+^, and P_soluble_ were also ameliorated when PNSB were used (Table 3). pH significantly affects the soil nutrient availability, growth, and yield of crops [31,32]. In acidic soils for canary melon, supplying the mixture of 4 P-solubilizing PNSB strains *Rhodopseudomonas palustris* VNW64, VNS89, TLS06, and VNW02 improved pH by 0.30–0.71 [33]. Likewise, supplying PNSB strains A3-5 and F3-3 increased the soil pH for rice [17]. Soil pH is vital due to its influence on the availability of nutrients in a rice-shrimp system [34]. Low pH increases Fe^2+^ and Al^3+^ toxicities and P precipitations and decreases N availability, leading to decreased plant growth [35,36]. Moreover, salinity can increase Na^+^ and Cl^−^ uptake, leading to lower uptake of essential nutrients such as N, P, K, Ca, and Mg [37]. PNSB can produce EPS to bind H^+^ because EPS has –OH and –COOH groups, leading to lower H^+^ concentration in the soil, i.e., pH increases [15]. The NH_4_^+^ concentration increases by the N_2_ fixation and soluble P increases by the solubilization of Ca-P, Al-P, and Fe-P by PNSB [24]. Moreover, phosphatase and phytase are also produced by *R. palustris* to solubilize inorganic P and increase soil pH [38]. Therefore, applying PNSB improves soil fertility and positively affects the P dynamics. Moreover, other soil parameters were also improved by the supplementation of PNSB. The EC dropped when supplying *C. sphaeroides* ST16 and ST26 (Table 2). This is in accordance with the study by Khuong et al. [24] where EC went down when *Luteovulum sphaeroides* W03 and W11 were supplied in rice cultivation. This can be explained by the fact that Na^+^ is fixed by galacturonic acid in EPS produced by PNSB [37]. The result can be evaluated according to the lower Na^+^ concentration in the treatment with PNSB than in the negative control (Table 3). The K^+^ concentrations in the treatments with the PNSB were improved (Table 3). As per Khuong et al. [39], PNSB can solubilize K under in vitro conditions. The K^+^ concentration was improved in saline soils and acidic saline soils for rice and soybean under the application of PNSB [24,40].

Based on the improvements in the soil nutrients, acidity, and salinity, the nutrient uptake of the rice plants was ameliorated. According to Table 3 and Figure 1, PNSB accumulated P in stem–leaf and grain, leading to greater total P uptake as compared with the case without bacteria. This follows a previous study where the mixture of *L. sphaeroides* can solubilize Al-P, Fe-P, and Ca-P present in the paddy soil, leading to greater total P uptake and reduced chemical fertilizer by 50% P according to the recommendation [24]. Table 4 and Figure 2 reveal that the Na content and uptake in stem–leaf and the total Na uptake in the treatments with *C. sphaeroides* varied in both seasons. The soil characteristic in the current study was considered acidic (pH 3.69–3.83) and salinized (EC 6.59) (Table 1). The peanut and rice root cannot control the Na uptake because rice is highly vulnerable to salinity [41,42], leading to great Na content in stem–leaf [43]. Supplying the mixture of PNSB can reduce soil Na content [14]. Furthermore, using *Rhodobacter* spp. can reduce NaCl by 28.57–36.42% on day 14 of incubation under 25 ppm NaCl [44]. Ultimately, from the reduction in soil salinity and acidity and the increases in soil nutrients, including soluble P, the uptake of rice plants followed the same patterns. This indicates the response of rice plants to the current biofertilizer.

Because the Na uptake in rice plants was reduced by the supplementation of the PNSB strains, the biochemical traits of rice plants changed. In treatments with *C. sphaeroides* ST16 and ST26, SPAD indices and contents of chlorophyll a, b, and a + b increased on day 35 after sowing in the first season and day 21 after sowing in the second season (Table 1). In rice farming, salinity interferes and reduces the chlorophyll content due to restrictions in nutrient availability [45]. However, PNSB can play a role as a biofertilizer to provide PGPS and nutrients (P, N, and K) [12,24,39]. Among the PGPS, ALA is the precursor of chlorophyll and can be produced by PNSB [12,46]. Therefore, supplying PNSB improved the N content and chlorophyll in leaves of crops, such as rice [47,48]. Ultimately, supplying PNSB promotes plant photosynthesis and improves rice growth. As compared with the negative control, the proline content in rice was lower in the treatments with PNSB in both seasons, except for the ST16 strain in the first season (Table 2). The proline content in plants, such as olive, grape, bean, and alfalfa, proportionally increases according to the salinity [49,50,51,52]. Thus, proline is one of the key indicators of plant response to salinity [49,50,51]. This result was consistent with the study by Khuong et al. [12], where supplying PNSB reduced proline in rice plants under a saline condition. This revealed that the salt stress on rice plants was lessened by the supplementation of the PNSB.

Following the improvements in plant biochemical traits, rice growth and yield were enhanced significantly. In brief, supplying PNSB resulted in greater growth (plant height and panicle length) and yield traits (panicle number pot^−1^, grain number panicle^−1^, and filled grain percentage) than the negative control. This resulted in a greater yield of 15.2–36.5% in the treatment with PNSB (Table 5, Figure 3). PNSB plays a role as a biofertilizer that can solubilize P and K and fix N, leading to greater P _soluble_, exchangeable K^+^, and available N for rice to absorb [12]. Moreover, PNSB can promote rice root [53] and produce PGPS such as ALA and EPS to reduce Na^+^ under saline stress to promote rice growth [24,54]. Therefore, PNSB increases rice tolerance under stresses, such as salinity and acidic salinity, to improve rice plant height, panicle length, yield traits, and rice grain yield in acidic, acidic–saline, and saline soils [12,16]. Additionally, applying the mixture of *L. sphaeroides* W011, W14, W22, and W32 increases yield up to 86.8% in saline soil [12]. Moreover, using both *Rhodopseudomonas palustris* BNCC134292 and *Bacillus subtilis* BNCC188062 improved rice yield by 9.84–17.73% [55]. For the rice variety, the OM5451 rice is moderately vulnerable to salinity, with reduced root length by 36% and plant height by 60% under saline irrigation [56]. However, under the application of the above PNSB strains, the performance of rice plants was very much improved compared to the treatments without PNSB at the same fertilizer levels (Figure 3). Thus, the above results show a promising vision of applying P-solubilizing PNSB to provide soluble P under saline conditions to ameliorate P dynamics, growth, and yield of rice.

## 5. Conclusions

Supplying *C. sphaeroides* ST16 and ST26 increased soluble P by 8.33–27.8% and decreased soil Na^+^ concentration by 5.87–55.0%, leading to increased total P uptake by 29.4–56.1% and decreased total Na uptake by 3.5–8.8%. From that, the biochemical traits, growth, and yield of rice plants were improved, leading to a greater rice grain yield by 15.2–36.5% compared with no bacteria used. Moreover, supplying the mixture of both *C. sphaeroides* ST16 and ST26 without N fertilizer showed a greater yield than the treatment with 100% P only. Therefore, it can be stated that 100% P fertilizers, as compared with the recommendation, can be replaced by the biofertilizer mixture. Moreover, the actual performance of the biofertilizer mixture should be further investigated under field conditions via continuous seasons.

## Figures and Tables

**Table 1:** Chemical characteristics of soil collected in An Bien–Kien Giang at a depth of 0–30 cm.

Soil Characteristic	Unit	Value	Status	Reference
pH_H2O_	_-_	3.82 ± 0.05	Low	Horneck et al. [20]
pH_KCl_	-	3.69 ± 0.08	Highly acidic	Horneck et al. [20]
EC	mS cm^−1^	6.59 ± 0.11	Only suitable for certain crops	Horneck et al. [20]
N_total_	%N	0.196 ± 0.09	Low	Metson [21]
NH_4_^+^	mg kg^−1^	49.8 ± 1.25	Optimum	Horneck et al. [20]
P_total_	%P_2_O_5_	0.021 ± 0.009	Poor	Cu et al. [22]
P_soluble_	mg kg^−1^	85.0 ± 3.22	High	Horneck et al. [20]
Al-P	mgkg^−1^	14.5 ± 0.22	-	-
Fe-P	mgkg^−1^	105.3 ± 2.24	-	-
Ca-P	mgkg^−1^	85.0 ± 4.65	-	-
CEC	meq 100 g^−1^	9.71 ± 0.20	Low	Landon [23]
Na^+^	meq 100 g^−1^	3.02 ± 0.08	High	Horneck et al. [20]
K^+^	meq 100 g^−1^	0.683 ± 0.05	High	Horneck et al. [20]
Ca^2+^	meq 100 g^−1^	3.07 ± 0.11	-	-
Mg^2+^	meq 100 g^−1^	2.82 ± 0.06	Extremely high	Horneck et al. [20]

Note: EC: electrical conductivity, N_total_: total nitrogen, NH_4_^+^: available nitrogen, P_total_: total phosphorus, P_soluble_: soluble P, Al-P: aluminum phosphate compounds, Fe-P: ferrous phosphate compounds, Ca-P: calcium phosphate compounds, CEC: cation exchange capacity, Na^+^: exchangeable sodium, K^+^: exchangeable potassium, Ca^2+^: exchangeable calcium, Mg^2+^: exchangeable magnesium. ± standard deviation.

**Table 2:** Phosphate fertilization and phosphate-solubilizing *Cereibacter sphaeroides* affected the chlorophyll and proline contents in rice in salinized soil in An Bien district, Kien Giang province.

Factor		SPAD				Chlorophyll			Proline
		21	28	35	42	a	b	A + b	(µmol g^−1^ DW)
		Days	Days	Days	Days	(µg g^−1^ Fresh Leaf Weight)			
Season 1									
Phosphate fertilizer percentage (A) (%)	0	39.2	38.8 ^b^	38.0 ^a^	37.9	4.78 ^b^	2.60 ^a^	7.50 ^a^	31.0 ^a^
	25	39.5	39.1 ^ab^	37.2 ^bc^	38.5	5.35 ^a^	2.44 ^b^	7.74 ^a^	29.8 ^b^
	50	39.3	38.8 ^b^	37.0 ^c^	38.6	5.17 ^a^	2.09 ^c^	7.21 ^b^	29.1 ^b^
	75	39.8	39.4 ^a^	37.7 ^ab^	38.4	5.14 ^a^	1.81 ^d^	6.88 ^c^	29.5 ^b^
	100	39.8	39.0 ^b^	37.6 ^ab^	38.4	5.21 ^a^	1.82 ^d^	7.03 ^bc^	29.4 ^b^
Phosphate-solubilizing bacteria (B)	NB	39.4 ^b^	38.9	36.9 ^b^	38.0 ^b^	4.18 ^c^	3.14 ^a^	7.31 ^b^	31.2 ^a^
	ST16	40.0 ^a^	39.0	36.6 ^b^	38.8 ^a^	5.72 ^a^	2.43 ^b^	8.15 ^a^	30.6 ^a^
	ST26	39.5 ^ab^	39.2	38.3 ^a^	38.6 ^ab^	5.67 ^a^	1.44 ^d^	7.17 ^b^	28.7 ^b^
	MIX	39.2 ^b^	39.0	38.1 ^a^	38.2 ^ab^	4.94 ^b^	1.59 ^c^	6.46 ^c^	28.5 ^b^
P (A)		ns	ns	*	ns	*	*	*	*
P (B)		*	*	*	*	*	*	*	*
P (A × B)		ns	ns	*	ns	*	*	*	ns
CV (%)		2.37	1.55	2.14	2.63	5.55	9.33	5.09	3.83
Season 2									
Phosphate fertilizer percentage (A) (%)	0	35.8 ^bc^	40.6	37.2	34.7 ^d^	3.56 ^b^	1.89 ^a^	5.45	34.8 ^a^
	25	36.5 ^a^	40.9	37.6	35.2 ^cd^	3.84 ^a^	1.76 ^b^	5.60	34.1 ^ab^
	50	35.3 ^d^	40.6	37.2	35.8 ^ab^	3.81 ^a^	1.73 ^b^	5.54	33.4 ^ab^
	75	36.0 ^b^	40.9	37.5	36.3 ^a^	3.78 ^a^	1.78 ^b^	5.56	34.3 ^ab^
	100	35.4 ^cd^	40.7	37.5	35.6 ^bc^	3.77 ^a^	1.69 ^b^	5.46	33.1 ^b^
Phosphate-solubilizing bacteria (B)	NB	34.9 ^c^	40.3 ^b^	36.9 ^b^	34.4 ^c^	3.49 ^c^	1.93 ^a^	5.42 ^b^	37.3 ^a^
	ST16	35.7 ^b^	41.0 ^a^	37.5 ^a^	35.1 ^b^	3.70 ^b^	1.71 ^b^	5.41 ^b^	32.5 ^b^
	ST26	37.2 ^a^	41.2 ^a^	37.6 ^a^	36.1 ^a^	4.03 ^a^	1.70 ^b^	5.72 ^a^	32.5 ^b^
	MIX	35.3 ^bc^	40.4 ^b^	37.5 ^a^	36.6 ^a^	3.79 ^b^	1.75 ^b^	5.54 ^b^	33.5 ^b^
P (A)		*	ns	ns	*	*	*	ns	*
P (B)		*	*	*	*	*	*	*	*
P (A × B)		*	ns	*	*	ns	*	*	*
CV (%)		1.87	1.83	2.08	2.14	5.27	7.12	4.11	5.49

Note: In the same column, numbers followed by different superscripted letters are different significantly; *: 5% significance level; ns: no significance; NB: no bacteria, ST16: *Cereibacter sphaeroides* ST16, ST26: *Cereibacter sphaeroides* ST26, and MIX: *Cereibacter sphaeroides* ST16 and ST26.

**Table 3:** Phosphate fertilization and phosphate-solubilizing *Cereibacter sphaeroides* affected the fertility of salinized soil in An Bien district, Kien Giang province, at harvesting.

Factors		pH_H2O_		pH_KCl_		CEC		Na^+^		K^+^		Mg^2+^		Ca^2+^	
		-		-		meq 100 g^−1^									
Season 1															
Phosphate fertilizer percentage (A) (%)	0	4.63		3.57		18.4		1.92 ^a^		1.534 ^e^		16.4 ^a^		3.27	
	25	4.71		3.60		18.8		1.84 ^b^		1.562 ^d^		16.1 ^ab^		3.32	
	50	4.74		3.64		18.4		1.84 ^b^		1.627 ^c^		15.7 ^abc^		3.35	
	75	4.51		3.54		19.1		1.74 ^c^		1.691 ^b^		15.4 ^c^		3.37	
	100	4.62		3.55		19.2		1.68 ^d^		1.794 ^a^		15.4 ^c^		3.37	
Phosphate-solubilizing bacteria (B)	NB	4.23 ^c^		3.54		18.1		1.84 ^a^		1.546 ^d^		16.0		3.24 ^b^	
	ST16	4.98 ^a^		3.61		19.0		1.78 ^b^		1.641 ^c^		15.8		3.36 ^a^	
	ST26	4.50 ^b^		3.54		19.1		1.80 ^ab^		1.662 ^b^		15.6		3.34 ^a^	
	MIX	4.87 ^a^		3.63		18.9		1.76 ^b^		1.718 ^a^		15.8		3.39 ^a^	
P (A)		ns		ns		ns		*		*		*		ns	
P (B)		*		ns		ns		*		*		ns		*	
P (A × B)		ns		ns		ns		*		*		ns		ns	
CV (%)		8.13		4.28		4.28		3.26		1.49		6.01		3.71	
Season 2															
Phosphate fertilizer percentage (A) (%)	0	5.53		3.87		16.9		0.145 ^a^		0.871 ^e^		15.3 ^d^		3.29 ^c^	
	25	5.54		3.89		16.0		0.127 ^b^		0.990 ^d^		16.3 ^c^		3.44 ^b^	
	50	5.64		3.99		16.0		0.113 ^c^		1.079 ^c^		19.3 ^b^		3.47 ^b^	
	75	5.53		3.84		16.8		0.100 ^d^		1.191 ^b^		20.3 ^a^		3.74 ^a^	
	100	5.59		4.07		16.8		0.093 ^d^		1.220 ^a^		20.5 ^a^		3.62 ^a^	
Phosphate -solubilizing bacteria (B)	NB	5.27 ^c^		3.82		16.0		0.172 ^a^		0.952 ^c^		18.0 ^c^		3.14 ^c^	
	ST16	5.83 ^a^		3.99		17.2		0.092 ^c^		1.056 ^b^		17.8 ^c^		3.62 ^b^	
	ST26	5.45 ^b^		3.92		16.5		0.093 ^c^		1.123 ^a^		18.5 ^b^		3.56 ^b^	
	MIX	5.71 ^a^		4.00		16.3		0.105 ^b^		1.149 ^a^		19.1 ^a^		3.74 ^a^	
P (A)		ns		ns		*		*		*		*		*	
P (B)		*		ns		*		*		*		*		*	
P (A × B)		ns		ns		*		*		*		*		ns	
CV (%)		4.27		7.35		11.12		9.47		4.14		1.46		4.91	
**Factors**		**EC**	**N _total_**		**P _total_**		**NH_4_^+^**		**P_soluble_**		**Fe-P**		**Ca-P**		**Al-P**
		**mS cm^−1^**	**%**				**mg kg^−1^**								
Season 1															
Phosphate fertilizer percentage (A) (%)	0	4.78 ^e^	0.215		0.043		231.1 ^c^		4.94 ^e^		151.3 ^d^		164.1 ^e^		140.9 ^d^
	25	4.88 ^d^	0.216		0.042		234.1 ^b^		5.29 ^d^		156.8 ^c^		171.7 ^d^		146.1 ^c^
	50	4.93 ^c^	0.224		0.044		235.4 ^b^		5.69 ^c^		162.7 ^b^		174.2 ^c^		148.8 ^b^
	75	4.97 ^b^	0.220		0.044		239.8 ^a^		5.87 ^b^		165.2 ^a^		179.7 ^b^		150.0 ^b^
	100	5.00 ^a^	0.218		0.042		241.4 ^a^		6.26 ^a^		167.0 ^a^		189.2 ^a^		158.0 ^a^
Phosphate -solubilizing bacteria (B)	NB	5.55 ^a^	0.218		0.043		228.5 ^d^		4.64 ^d^		174.4 ^a^		193.5 ^a^		164.4 ^a^
	ST16	5.06 ^b^	0.216		0.043		234.5 ^c^		5.71 ^c^		166.8 ^b^		174.6 ^b^		152.7 ^b^
	ST26	4.56 ^c^	0.223		0.044		238.4 ^b^		5.92 ^b^		161.4 ^c^		169.2 ^c^		140.9 ^c^
	MIX	4.49 ^d^	0.217		0.043		244.1 ^a^		6.16 ^a^		139.8 ^d^		165.8 ^d^		137.1 ^d^
P (A)		*	ns		ns		*		*		*		*		*
P (B)		*	ns		ns		*		*		*		*		*
P (A × B)		ns	ns		ns		*		*		*		*		*
CV (%)		0.67	7.85		8.36		1.01		3.92		1.85		1.45		2.15
Season 2															
Phosphate fertilizer percentage (A) (%)	0	4.26 ^e^	0.201		0.038		119.2 ^c^		27.4 ^e^		118.0 ^e^		66.3 ^e^		16.3 ^c^
	25	4.35 ^d^	0.201		0.036		124.5 ^b^		28.2 ^d^		120.6 ^d^		67.7 ^d^		16.6 ^c^
	50	4.47 ^c^	0.210		0.038		125.7 ^b^		28.7 ^c^		122.5 ^c^		69.7 ^c^		17.2 ^b^
	75	4.52 ^b^	0.193		0.036		128.2 ^a^		29.1 ^b^		127.8 ^b^		76.1 ^b^		17.4 ^b^
	100	4.62 ^a^	0.199		0.038		130.4 ^a^		31.0 ^a^		130.1 ^a^		81.3 ^a^		20.6 ^a^
Phosphate -solubilizing bacteria (B)	NB	5.31 ^a^	0.205		0.038		115.3 ^d^		27.2 ^d^		133.9 ^a^		78.3 ^a^		26.0 ^a^
	ST16	4.28 ^b^	0.209		0.036		123.6 ^c^		27.9 ^c^		125.9 ^b^		76.0 ^b^		17.8 ^b^
	ST26	4.13 ^c^	0.200		0.036		129.5 ^b^		29.3 ^b^		119.5 ^c^		72.6 ^c^		14.9 ^c^
	MIX	4.06 ^d^	0.190		0.038		133.9 ^a^		31.2 ^a^		115.9 ^d^		62.0 ^d^		11.7 ^d^
P (A)		*	ns		ns		*		*		*		*		*
P (B)		*	ns		ns		*		*		*		*		*
P (A × B)		*	ns		ns		*		*		*		*		*
CV (%)		1.30	15.04		10.29		2.68		1.20		1.99		2.40		3.27

Note: In the same column, numbers followed by different superscripted letters are different significantly; *: 5% significance level; ns: no significance; NB: no bacteria, ST16: *Cereibacter sphaeroides* ST16, ST26: *Cereibacter sphaeroides* ST26, and MIX: *Cereibacter sphaeroides* ST16 and ST26.

**Table 4:** Phosphate fertilization and phosphate-solubilizing *Cereibacter sphaeroides* affected the biomass and Na-P content and uptake in rice plants in salinized soil in An Bien district, Kien Giang province.

Factor		Biomass		Na Content		Na Uptake		Total Na Uptake	P Content		P Uptake		Total P Uptake
		Stem–Leaf	Grain	Stem–Leaf	Grain	Stem–Leaf	Grain		Stem–Leaf	Grain	Stem–Leaf	Grain	
		g pot ^−1^		%		mg Na pot^−1^			%		mg P pot^−1^		
**Season 1**													
Phosphate fertilizer percentage (A) (%)	0	12.0 ^e^	15.1 ^e^	1.63 ^a^	1.15 ^a^	203.9 ^a^	198.4 ^a^	402.4 ^a^	0.188 ^a^	0.281 ^a^	21.7 ^c^	42.6 ^b^	65.1 ^cd^
	25	12.4 ^d^	15.9 ^d^	1.55 ^b^	1.13 ^ab^	201.3 ^b^	191.8 ^b^	393.0 ^b^	0.176 ^b^	0.252 ^b^	22.5 ^c^	40.6 ^b^	62.3 ^d^
	50	13.0 ^c^	16.9 ^c^	1.52 ^c^	1.12 ^ab^	196.1 ^c^	190.5 ^b^	386.6 ^c^	0.192 ^a^	0.245 ^bc^	22.5 ^c^	42.0 ^b^	67.1 ^bc^
	75	13.5 ^b^	19.4 ^b^	1.54 ^bc^	1.11 ^b^	195.2 ^c^	186.7 ^c^	381.9 ^d^	0.167 ^c^	0.238 ^bc^	25.1 ^b^	46.9 ^a^	69.3 ^b^
	100	14.2 ^a^	20.8 ^a^	1.54 ^bc^	1.10 ^b^	195.3 ^c^	186.4 ^c^	381.7 ^d^	0.189 ^a^	0.235 ^c^	26.8 ^a^	48.8 ^a^	75.6 ^a^
Phosphate -solubilizing bacteria (B)	NB	11.8 ^d^	11.8 ^d^	1.62 ^a^	1.21 ^a^	205.3 ^a^	197.8 ^a^	403.2 ^a^	0.184 ^b^	0.222 ^b^	21.6 ^c^	26.1 ^c^	47.8 ^d^
	ST16	12.8 ^c^	18.7 ^c^	1.56 ^b^	1.13 ^b^	197.9 ^b^	191.1 ^b^	389.0 ^b^	0.177 ^c^	0.257 ^a^	22.7 ^b^	47.7 ^b^	70.4 ^cd^
	ST26	13.3 ^b^	19.1 ^b^	1.54 ^b^	1.07 ^c^	195.6 ^c^	190.0 ^b^	385.6 ^c^	0.193 ^a^	0.260 ^a^	25.5 ^a^	48.3 ^b^	73.9 ^b^
	MIX	14.3 ^a^	20.9 ^a^	1.50 ^c^	1.08 ^c^	194.7 ^c^	184.2 ^c^	378.8 ^d^	0.175 ^c^	0.262 ^a^	25.0 ^a^	54.4 ^a^	79.5 ^a^
P (A)		*	*	*	*	*	*	*	*	*	*	*	*
P (B)		*	*	*	*	*	*	*	*	*	*	*	*
P (A × B)		*	*	*	*	*	*	*	*	*	*	*	*
CV (%)		3.03	2.61	2.43	4.21	1.32	1.67	1.07	5.82	7.89	6.39	8.48	6.39
**Season 2**													
Phosphate fertilizer percentage (A) (%)	0	21.4 ^e^	20.5 ^e^	0.766 ^a^	0.722 ^a^	164.1 ^a^	146.5 ^a^	310.5 ^a^	0.140 ^d^	0.141 ^c^	30.1 ^d^	28.9 ^e^	59.0 ^d^
	25	21.9 ^d^	21.6 ^d^	0.731 ^b^	0.585 ^b^	159.9 ^bc^	125.9 ^b^	285.8 ^bc^	0.158 ^c^	0.141 ^bc^	34.8 ^c^	30.7 ^d^	65.5 ^c^
	50	23.0 ^c^	22.5 ^c^	0.664 ^c^	0.544 ^bc^	151.9 ^d^	121.8 ^b^	273.7 ^c^	0.169 ^ab^	0.150 ^a^	38.8 ^b^	33.8 ^c^	72.7 ^b^
	75	24.1 ^b^	24.0 ^b^	0.653 ^c^	0.491 ^c^	156.6 ^c^	117.3 ^b^	273.8 ^c^	0.164 ^bc^	0.149 ^a^	39.9 ^b^	35.7 ^b^	75.6 ^b^
	100	24.9 ^a^	25.7 ^a^	0.655 ^c^	0.518 ^c^	162.2 ^ab^	132.0 ^b^	294.2 ^b^	0.174 ^a^	0.146 ^ab^	43.7 ^a^	37.6 ^a^	79.2 ^a^
Phosphate-solubilizing bacteria (B)	NB	21.4 ^d^	20.7 ^d^	0.766 ^a^	0.699 ^a^	163.9 ^a^	143.7 ^a^	307.6 ^a^	0.138 ^b^	0.135 ^b^	29.6 ^c^	28.0 ^c^	57.7 ^c^
	ST16	22.5 ^c^	22.5 ^c^	0.679 ^b^	0.576 ^b^	152.4 ^c^	128.2 ^b^	280.6 ^b^	0.168 ^a^	0.148 ^a^	37.7 ^b^	33.4 ^b^	71.1 ^b^
	ST26	23.8 ^b^	23.6 ^b^	0.688 ^b^	0.512 ^c^	162.7 ^a^	118.9 ^b^	281.6 ^b^	0.172 ^a^	0.151 ^a^	41.3 ^a^	35.7 ^a^	77.1 ^a^
	MIX	24.6 ^a^	24.7 ^a^	0.641 ^c^	0.501 ^c^	156.7 ^b^	123.9 ^b^	280.6 ^b^	0.166 ^a^	0.147 ^a^	41.2 ^a^	36.3 ^a^	75.8 ^a^
P (A)		*	*	*	*	*	*	*	*	*	*	*	*
P (B)		*	*	*	*	*	*	*	*	*	*	*	*
P (A × B)		*	*	*	*	*	*	*	*	*	*	*	*
CV (%)		1.17	1.25	3.02	14.2	3.15	15.11	6.96	5.48	4.90	5.82	5.52	6.56

Note: In the same column, numbers followed by different superscripted letters are different significantly; *: 5% significance level; NB: no bacteria, ST16: *Cereibacter sphaeroides* ST16, ST26: *Cereibacter sphaeroides* ST26, and MIX: *Cereibacter sphaeroides* ST16 and ST26.

**Table 5:** Phosphate fertilization and phosphate-solubilizing *Cereibacter sphaeroides* affected the growth, yield traits, and yield of rice plants in salinized soil in An Bien district, Kien Giang province.

Factor		Plant Height	Panicle Length	Panicle Number pot^−1^	Grain Number Panicle^−1^	1000-Grain Weight	Filled Grain Percentage	Yield
		(cm)		(panicles)	(grains)	(g)	(%)	(g pot^−1^)
**Season** 1								
Phosphate fertilizer percentage (A) (%)	0	88.8 ^e^	20.4	18.3 ^e^	77.5	21.9	66.2 ^e^	16.1 ^e^
	25	91.0 ^d^	20.0	20.2 ^d^	66.5	21.7	67.2 ^d^	17.3 ^d^
	50	92.2 ^c^	20.0	21.0 ^c^	67.7	21.8	70.8 ^c^	18.8 ^c^
	75	93.9 ^b^	20.6	22.0 ^b^	74.6	22.1	73.8 ^b^	20.0 ^b^
	100	95.0 ^a^	21.0	23.0 ^a^	76.8	22.1	77.5 ^a^	21.6 ^a^
Phosphate-solubilizing bacteria (B)	NB	86.8 ^d^	19.1 ^b^	16.9 ^d^	60.1 ^b^	21.8	66.4 ^d^	14.7 ^d^
	ST16	92.4 ^c^	20.7 ^a^	20.5 ^c^	75.2 ^a^	22.2	70.8 ^c^	18.3 ^c^
	ST26	93.4 ^b^	20.8 ^a^	22.5 ^b^	75.2 ^a^	21.7	71.9 ^b^	19.7 ^b^
	MIX	96.1 ^a^	20.9 ^a^	23.6 ^a^	80.0 ^a^	22.0	75.2 ^a^	22.2 ^a^
P (A)		*	ns	*	ns	ns	*	*
P (B)		*	*	*	*	ns	*	*
P (A × B)		*	ns	*	ns	ns	*	*
CV (%)		1.33	6.37	2.95	20.60	3.11	1.14	3.01
**Season 2**								
Phosphate fertilizer percentage (A) (%)	0	89.3 ^d^	18.8	18.5 ^e^	66.9	22.9	89.4 ^d^	20.9 ^e^
	25	89.8 ^c^	18.7	19.0 ^d^	65.2	23.0	90.5 ^c^	21.5 ^d^
	50	91.1 ^b^	18.4	19.6 ^c^	63.9	23.3	90.8 ^c^	23.9 ^c^
	75	91.5 ^b^	18.9	20.2 ^b^	65.8	23.4	91.2 ^b^	24.3 ^b^
	100	92.4 ^a^	19.0	21.4 ^a^	64.1	23.6	92.5 ^a^	27.0 ^a^
Phosphate-solubilizing bacteria (B)	NB	88.9 ^d^	18.6	17.2 ^d^	65.4	23.1	87.4 ^d^	21.1 ^d^
	ST16	90.8 ^c^	19.2	19.6 ^c^	66.3	23.1	90.8 ^c^	22.6 ^c^
	ST26	91.4 ^b^	18.7	20.6 ^b^	65.4	23.4	91.2 ^b^	23.6 ^b^
	MIX	92.0 ^a^	18.7	21.5 ^a^	63.6	23.4	94.1 ^a^	26.7 ^a^
P (A)		*	ns	*	ns	ns	*	*
P (B)		*	ns	*	ns	ns	*	*
P (A × B)		*	ns	*	ns	ns	*	*
CV (%)		0.69	4.87	2.49	9.86	3.66	0.60	1.88

Note: In the same column, numbers followed by different superscripted letters are different significantly; *: 5% significance level; ns: no significance; NB: no bacteria, ST16: *Cereibacter sphaeroides* ST16, ST26: *Cereibacter sphaeroides* ST26, and MIX: *Cereibacter sphaeroides* ST16 and ST26.

## Data Availability

The original contributions presented in this study are included in the article. Further inquiries can be directed to the corresponding author.

## Abbreviations

The following abbreviations are used in this manuscript:

ALA	5-aminolevulinic acid
Al-P	Aluminum phosphate
Ca-P	Calcium phosphate
CEC	Cation exchange capacity
CFU	Colony forming unit
Cl	Chloride
EC	Electrical conductivity
EPS	Exopolymeric substances
Fe-P	Ferrous phosphate
IAA	Indole-3-acetic acid
Na	Sodium
P	Phosphate
PGPS	Plant growth-promoting substances
PNSB	Purple nonsulfur bacteria
SPAD	Soil and Plant Analysis Development
